# Supplementary material for: Enterococcus faecalis Countermeasures Defeat a Virulent Picovirinae Bacteriophage
Source: Viruses. 2019 Jan 10;11(1):48. doi: 10.3390/v11010048 (PMC6356687; doi:10.3390/v11010048)
Supplement: Supplementary file 1 [file viruses-11-00048-s001.pdf]

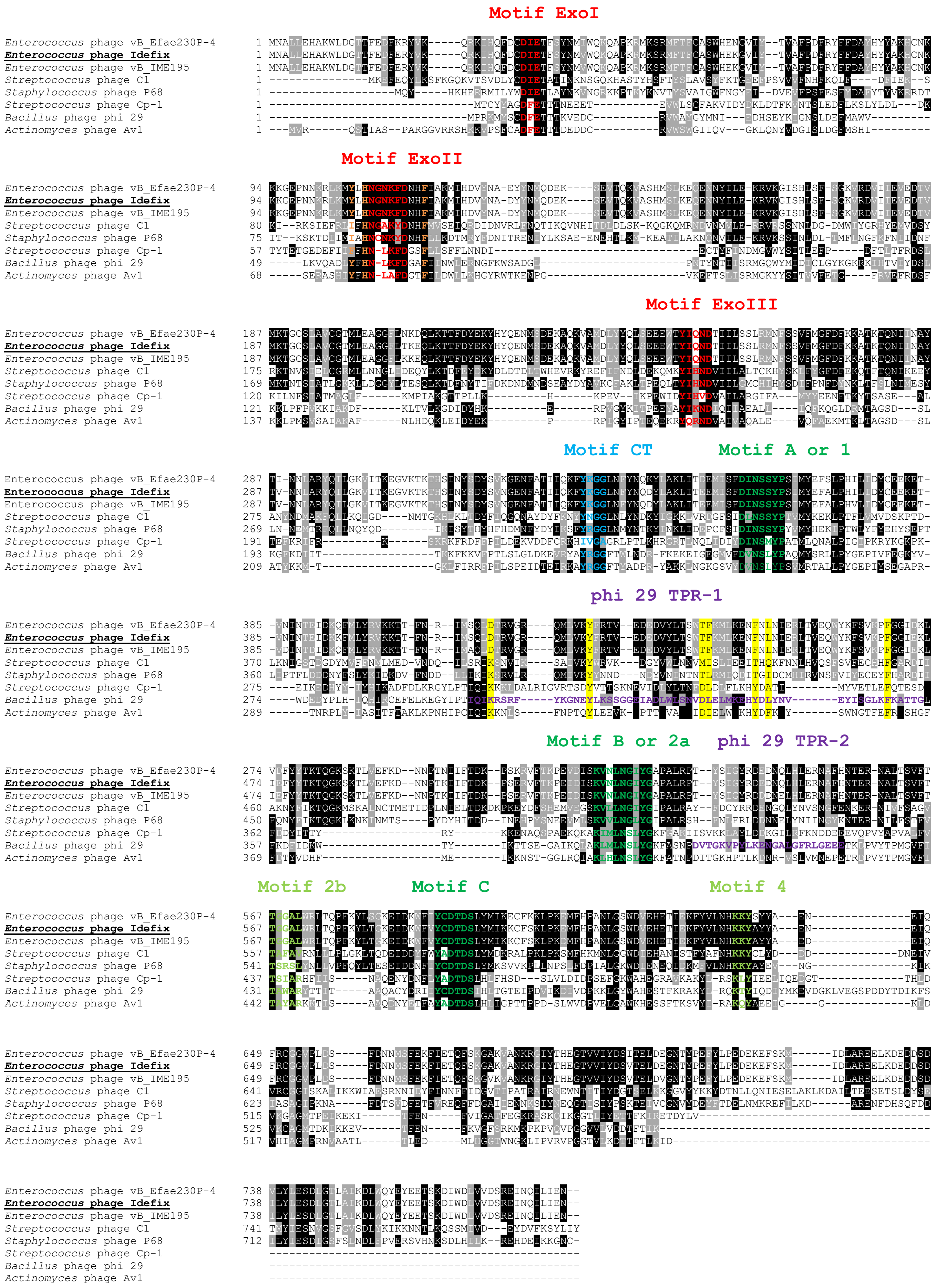
Motif ExoI
Motif ExoII
Motif ExoIII
Motif CT
Motif A or 1
phi 29 TPR-1
Motif B or 2a
phi 29 TPR-2
Motif 2b
Motif C
Motif 4
Enterococcus phage vB_Efae230P-4
Enterococcus phage Idefix
Enterococcus phage vB_IME195
Streptococcus phage C1
Staphylococcus phage P68
Streptococcus phage Cp-1
Bacillus phage phi 29
Actinomyces phage Av1

Figure S1: Structural and functional map of family of B phage DNA polymerases belonging to the "protein priming" subfamily. Alignments of the residues of *Enterococcus* phage VB_Efae230P-4, *Enterococcus* phage Idefix, *Enterococcus* phage VB_IME195, *Streptococcus* phage C1, *Staphylococcus* phage P68, *Streptococcus* phage Cp-1, *Bacillus* phage phi 29 and *Actinomyces* phage Av1 DNA polymerases are shown. Three N-terminally located motifs ExoI, ExoII and ExoIII (marked in red) constitute the 3'-5' exonuclease active site which is conserved in eukaryotic and prokaryotic polymerases [67,70]. The motif ExoII additionally presents 3 conserved residues (marked in orange) involved in the interaction with the Terminal Protein [69]. The N-terminal domain is separated from the C-terminal domain by a cross-talk conserved motif CT (marked in blue) playing an important role in the coordination between the previously mentioned proofreading activity and the synthesis activity performed in the C-terminal domain. Three C-terminally consensus motifs A, B, and C (marked in dark green) form the polymerase active site that is conserved in the other polymerases from the B family [67,70]. Two additional motifs 2b and 4 (marked in light green) notably involved in the primer stabilization are found in several B DNA polymerases. The subfamily of protein-priming DNA polymerases specifically harbor two inserted sequences TPR-1 and TPR-2, interspersed between conserved motifs A and B and B and C respectively. These 2 sequences have notably been studied in phi 29 DNA polymerase (marked in purple) and demonstrated as involved in Terminal Protein interaction and both strand displacement and processivity respectively [68,71]. Whether the 2 inserted sequences are not conserved among the protein priming subfamily of B DNA polymerase, several identical residues (underlined in yellow) are found in TPR-1 [68].

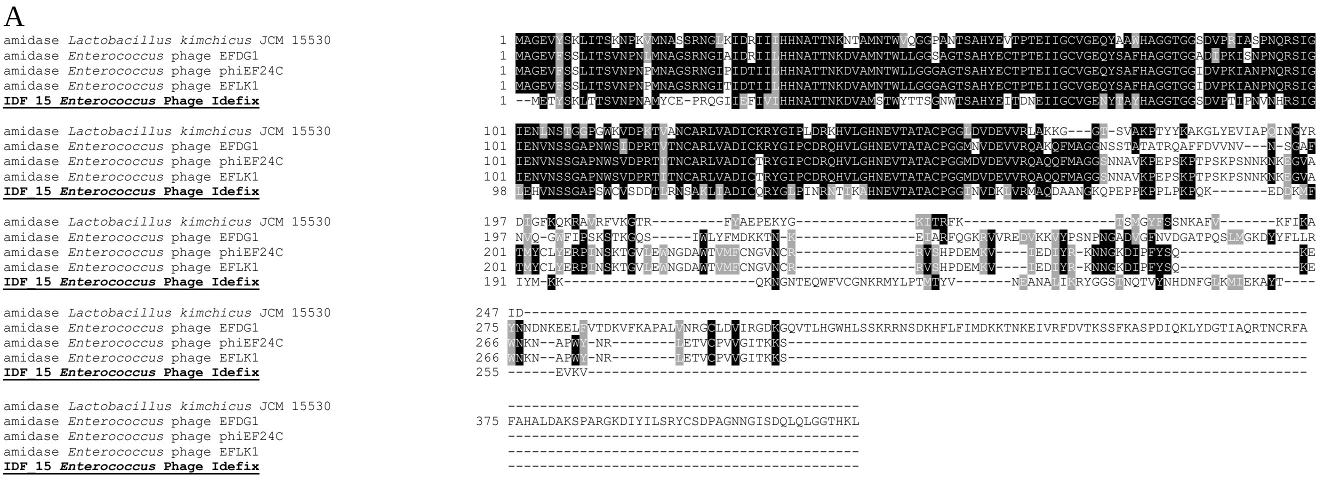

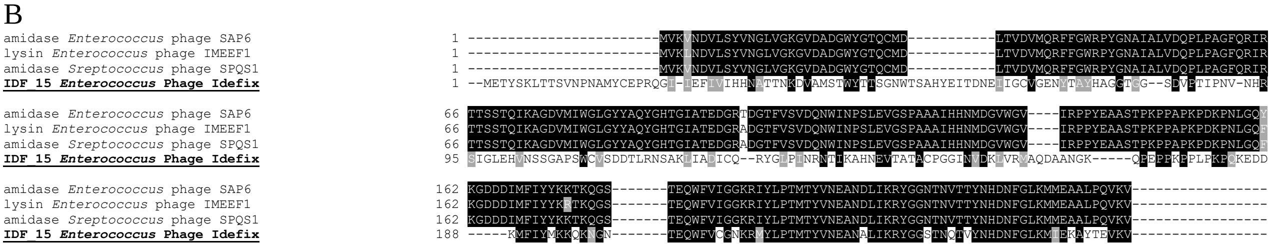

Figure S2: IDF_15 alignments. (**A**) Alignments of residues of IDF_15 with amidases with similar N terminal domains identified by BLASTP**.** IDF_15 N-terminal domain (residues 21-153) is predicted to correspond to a N-acetylmuramoyl-L-alanine amidase domain on pfam (http://pfam.xfam.org/). IDF_15 shares ~ 60% amino acids identity and ~ 60-75% coverage (N terminal terminuses) with amidases encoded by a *Lactobacillus kimchicus* strain and *Enterococcus* phages EFDG1, phiEF24C and EFLK1. (**B**) Alignments of residues of IDF_15 with hydrolases with similar C terminal domains identified by BLASTP. IDF_15 C shares ~ 70 % amino acids identity and ~ 40 % coverage (C-terminuses) with amidases and lysin encoded by an *Enterococcus* phage SAP6, *Streptococcus* phage SPQS1 and *Enterococcus* phage IMEEF1 respectively.

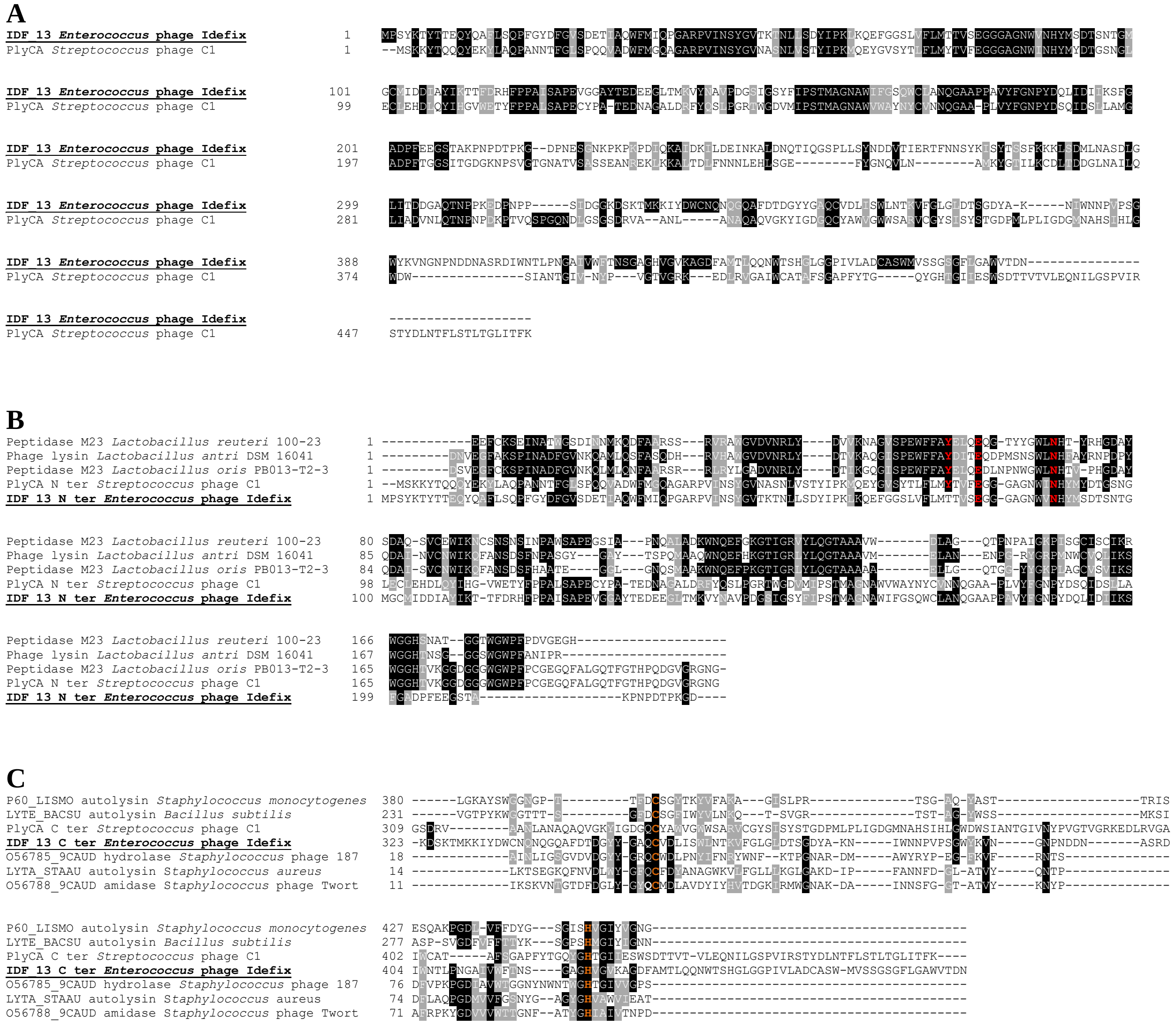

Figure S3: Alignments of IDF_13. (**A**) Alignment of the residues of IDF_13 and those of the catalytic subunit PlyCA encoded by *Streptococcus* phage C1. BLASTP indicates that the two proteins share 37 % amino acids identity and 77 % coverage. (**B**) Alignments of the residues of IDF_13 N-terminal domain with glycosyl hydrolases with similar domains identified by PSI-BLAST (modified from [75]). PlyCA N terminal domain (residues 1-205) corresponds to a glycosyl hydrolase domain homologous to those found in peptidases M23 and phage lysin encoded by *Lactobacillus reuteri*, *oris* and *antri* respectively. IDF_13 N-terminal domain shares 55 % amino acids identity and 97 % coverage with PlyCA N terminal domain, it notably harbors two out of the three conserved residues (marked in red) which are supposed to represent a catalytic center [75]. (**C**) Alignments of the residues of IDF_13 C-terminal domain with CHAP domains identified by PSI-BLAST (modified from [74,76]). PlyCA C-terminal domain (residues 309 to 465) corresponds to a CHAP domain with an *N*-acetylmuramoyl-L-alanine amidase activity [75]. The C-terminuses of IDF_13 and PlyCA are less conserved than the N-terminuses. Nevertheless, analyses reveal that IDF_13 C-terminal domain shares similarity with CHAP domains identified in autolysins encoded by *Staphylococcus monocytogenes*, *Staphylococcus aureus* and *Bacillus subtilis* or amidase and hydrolase encoded by *Staphylococcus* phages Twort and 187 respectively. IDF_13 C-terminal domain harbors the two predicted catalytic residues (marked in orange) which characterized CHAP domains [74,76].

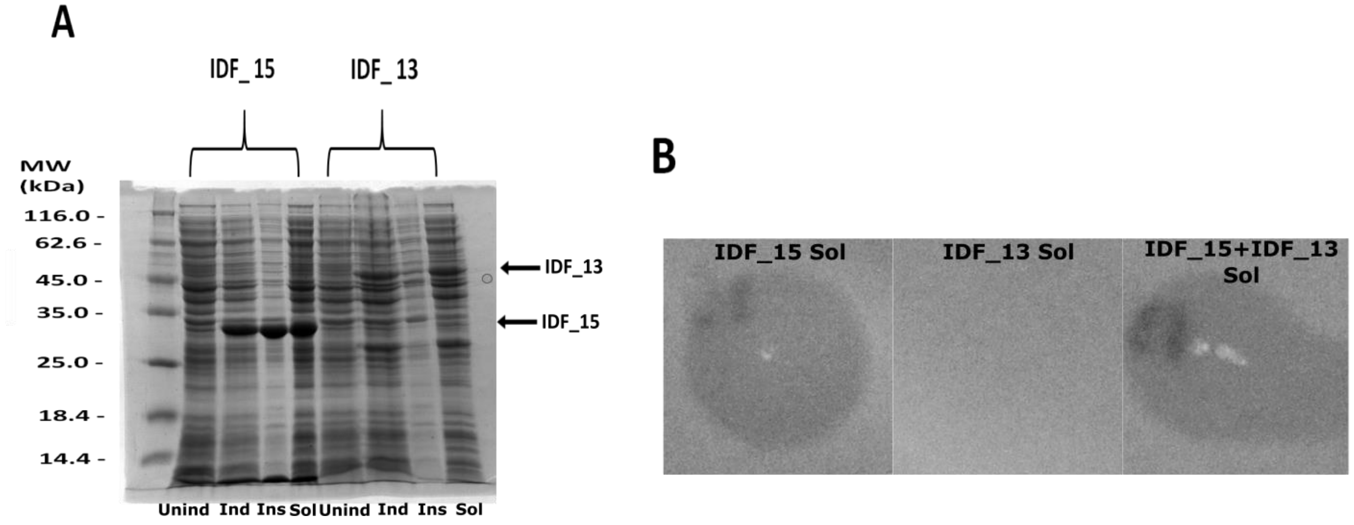

Figure S4 : Activities of *E.coli* extracts containing putative Idefix endolysins (IDF_15 and IDF_13). (**A**) Production of IDF_15 (29.6 kDa) and IDF13 (52.2 kDa) in *E. coli* ER2566 transformed by pAB2 and pAB1 respectively were assessed by analysis of 10 µL of harvested induced (ind) or uninduced (unind) cells resuspended in lysis buffer (10X concentration compared to the cell culture). After lysis and centrifugation, 2 µL of soluble (sol) and insoluble (insol) extracts (50X concentration compared to the cell culture) of uninduced or induced cells were analyzed by SDS-PAGE (12.5%) and coomassie blue staining, revealing an induced protein in the soluble fraction, of a size compatible with IDF_15 and IDF_13, respectively. (**B**) Evaluation of IDF_15 and IDF13 activities on pp^{-}, 5 µL of soluble extracts of induced cells containing IDF_15 or IDF13 and 5 µL of a mix of the two previous fractions were respectively spotted on a pp^{-} bacterial lawn.

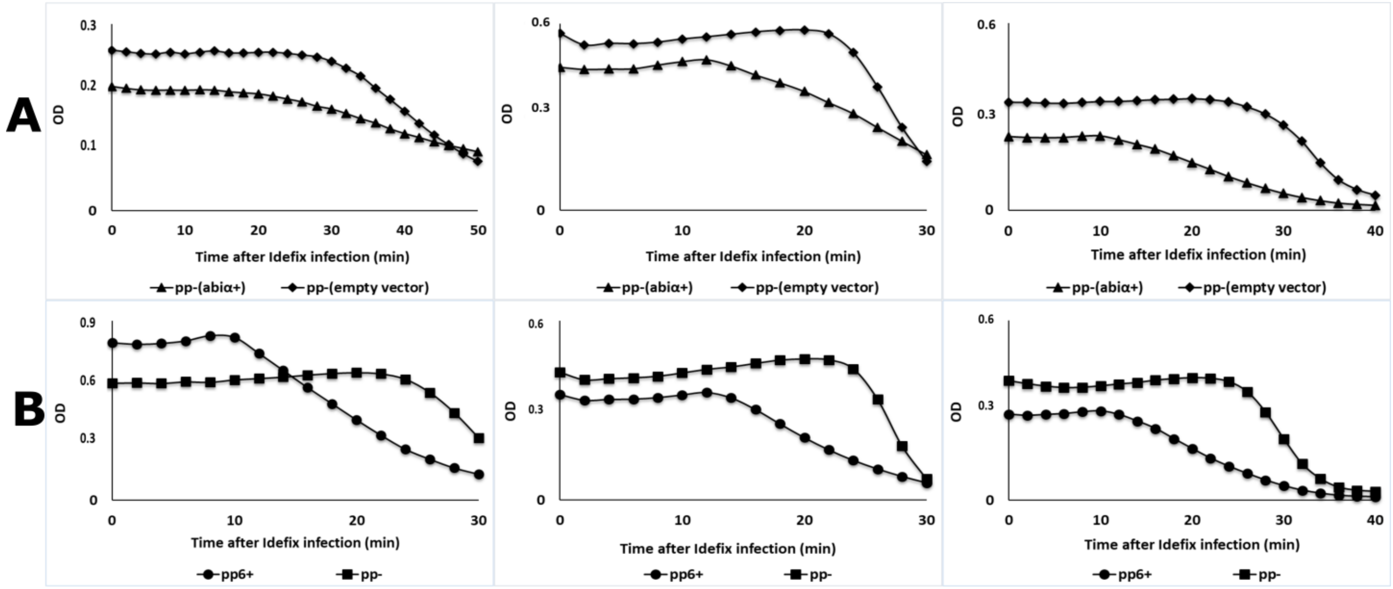

Figure S5 : Biological replicates relative to lysis curves experiments after Idefix infection at MOI 10. (**A**) Comparison between pp-pJIM2246abiα^+ (VEJL5) and pp- pJIM2246 (VEJL4) in triangles and diamonds, respectively (three independent experiments). (**B**) Comparison between pp6+ and pp- in circles and squares, respectively (three independent experiments).

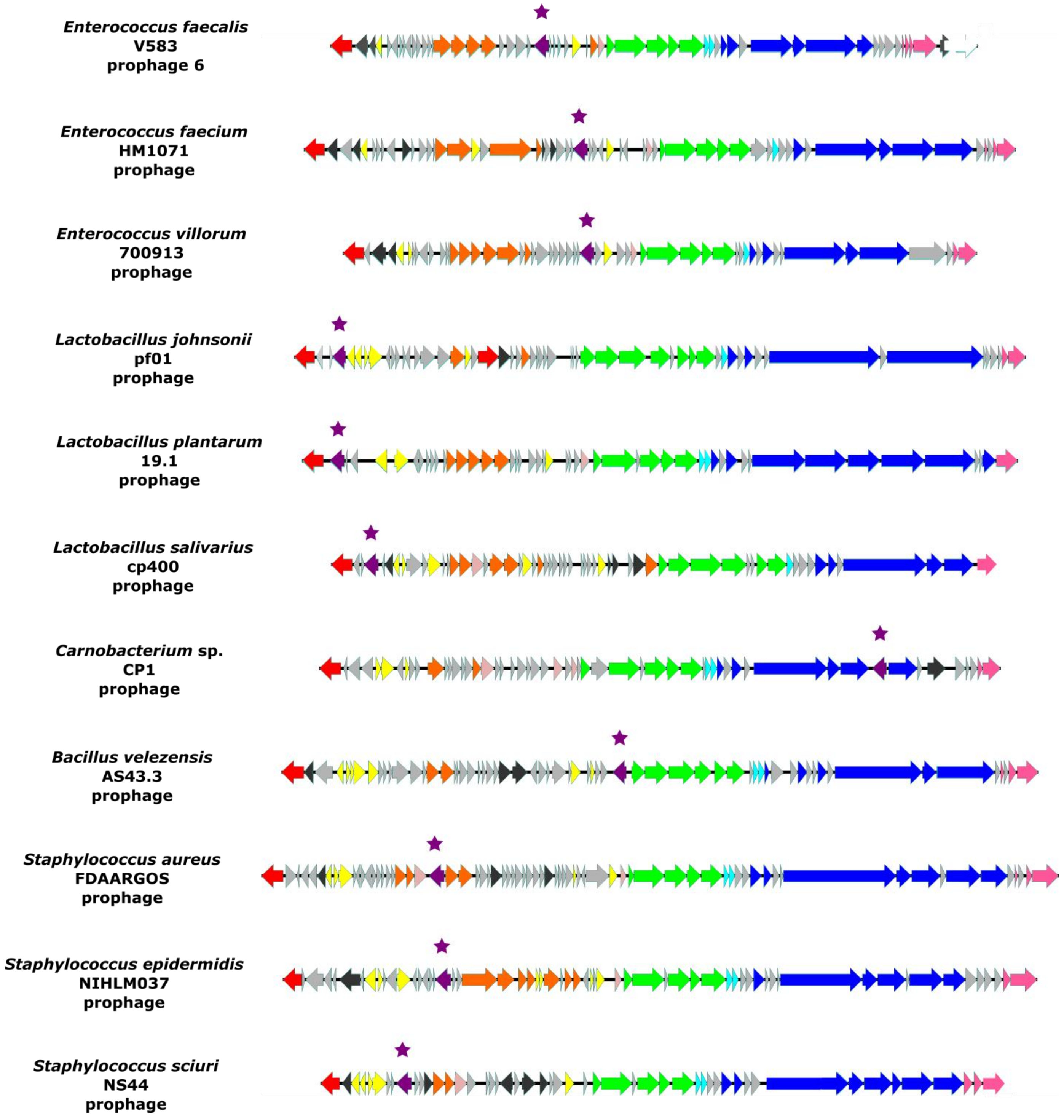

Figure S6: Putative *abiα* homologs identified on several prophages of Gram-positive bacteria. *abiα* and its putative homologs are colored in purple and marked with stars. Other gene functions are color-coded (red: integrase, yellow: transcriptional regulation, orange: DNA metabolism, green: DNA packaging and head, light blue: head to tail, dark blue: tail, light pink: HNH endonuclease, dark pink: lysis, grey: hypothetical proteins, white: transposable element, black: additional functions including putative anti-phage systems).

Table S1: Bacterial strains, plasmids and oligonucleotide primers used

Strain, plasmid or primer	Relevant characteristics or primer sequences	Reference or source
Strain		
E. coli		
JM105	*endA1 glnV44 sbcB15 rpsL thi*-1 Δ(*lac-proAB*) [F' *traD36* proAB$^+$ *lacI*^{q} *lacZ*ΔM15] *hsdR4*(r_K^- m_K^+)	NEB
ER2566	F$^-$ λ^- *fhuA2 ompT lacZ*::T7pol *gal sulA11 Δ*(*mcrC-mrr*)114 *mcr*-73 *zgb*-210 *endA1*	NEB
L. lactis		
MG1653	Genetically amenable plasmid-free strain	[46]
E. faecalis		
VE14002	V583 vancomycin resistant clinical isolate	[50]
VE14089	V583 vancomycin resistant clinical isolate cured of its plasmids	[48]
VE18590	VE14089 derivative deleted from all its plasmids and active prophages	[43]
VE18562	VE14089 derivative deleted from all its plasmids but pp1	[43]
VE18584	VE14089 derivative deleted from all its plasmids but pp3	[43]
VE18583	VE14089 derivative deleted from all its plasmids but pp3 and pp5	[43]
VE18582	VE14089 derivative deleted from all its plasmids but pp4	[43]
VE18589	VE14089 derivative deleted from all its plasmids but pp7	[43]
VE18581	VE14089 derivative deleted from all its plasmids but pp6	[43]
VE18306	VE14089 derivative only deleted from pp6	[43]
VEJL1	VE18306 derivative complemented with pJL1	This study
VEJL2	VE18306 derivative complemented with pJL2	This study
VEJL3	VE18306 derivative complemented with pJL3	This study
VEJL4	VE18590 derivative complemented with pJIM2246	This study
VEJL5	VE18590 derivative complemented with pJL3	This study
VE18393	VE18306 derivative deleted from *epaX* (*ef2170*)	This study
VE18944	VE18393 derivative complemented with pVE14176	This study
VE18945	VE18393 derivative complemented with pVE14297	This study
Plasmid		
pJIM2246	*E. coli*-Gram positive shuttle vector Cm^r	[47]
pJL1	*ef2850* cloned in pJIM2246	This study
pJL2	*ef2847* cloned in pJIM2246	This study
pJL3	*ef2833* cloned in pJIM2246	This study
pVE14283	encompassing 5' and 3' ends *epaX* insert cloned in pG+host9	[49]
pVE14176	promoter PaphA3 vector made of pIL253 and pVE14041	[45]
pVE14297	*epaX* cloned downstream promoter PaphA3 in pVE14176	[49]
pJ411	PBR322 origin, kanR, pT7	Menlo Park CA
pAB1	*IDF_13* cloned in pJ411	This study
pAB2	*IDF_15* cloned in pJ411	This study
Primer		
JL12	TACGGATCCTTGAACAGCGGAACG	This study
JL13	AATGAGCTAGGGAGTGGAGTTTG	This study
JL10	TACGGATCCCTATCACCTCCTATAC	This study
JL11	TACGGATCCTTATCAAAG	This study
JL8	TACGGATCCTTAGCGCTGCCCTTAC	This study
JL9	AATGTCGACTG GCGAGTGACTGG	This study
JL20	ATGCGCGCGTTGTTTTAAATAATTGGAG	This study
JL21	ATCGCATGCGCATGATATTACTCTCCTAC	This study
OEF879	GGATTGGATTAGTTCTTGTG	Furlan et al, submitted
1233	AGCGGATAACAATTTCACACAGGA	NEB
OEF528	TCATTCTTCTCCCAAGCTTC	[48]
OEF880	GCGACAATGATTGTATTTGC	This study
OEF394	AGTTATCGGAGATGTCACAG	[49]
OEF397	ATCACCATTGCACAAACCAC	[49]
OEF527	TGCTCGTAATGCTGGAATTG	[48]
OEF856	CTTTATTCTGTTCACAAGTGG	Furlan et *al*., submitted
OEF823	TAGGGCCCAAGTAATGTTGATGAGAACG	Furlan et *al*., submitted
OEF859	AATTATCGGTTTTTCCTCTACC	Furlan et *al*., submitted
OEF857	AAATCTCGTCCTCCTAATGC	Furlan et *al*., submitted
OEF885	TACACGTATTTTCAGGATTGG	This study
OEF858	ATCTATACGCATCTGAAATGG	Furlan *et al*., submitted
OFL264	AATTCATATGCCTAGTTACAAAACTTATAC	This study
OFL265	AATTGGATCCCTAATTATCTGTAACCCAAG	This study
OFL266	AATTCATATGGAAACCTACTCAAAATTAACAAC	This study
OFL267	AATTGGATCCTTATACTTTAACTTCCGTATAAG	This study

Table S2. Description of natural *Enterococcus* isolates tested as potential Idefix hosts

E. faecalis						
**Isolate (Other name)**	**Origin[a]**	**Source; origin; year of isolation**	**Ab[R b]**	**ST[c]**	**CPS[d]**	**CRISPRs[e]**
VE14001 (OG1RF)	C	Mouth; USA; 1978	F; R	1	T1	+/+
VE14002 (V583)	C	Blood; Missouri, USA; 1987	E; G; K; V	6	T2	-/+
VE14000 (JH2-2)	C	Hammersmith, UK; 1973	F; R	8	T2	-/+
VE14568 (G51)	C	Endocarditis; USA; <1992	M; T	19	T1	+/+
VE14569 (G52)	C	Endocarditis; USA; <1992	E; K; M; T	19	T1	+/+
VE14571 (G54)	Cm	Human stool; Oklaoma, USA; 1994	M; T	19	T1	+/+
VE14840 (F11/KA1.2)[f]	F	Cheese; France; 2005	C; E; K; M; T	21	T1	-/+
VE14741 (efs2)	F	Cheese (St Paulin); France; 1991	M; T	72	T5	-/+
VE14633 (1085)	F	Cheese (Salers); Cantal, France; 1991	-	277	T2	-/+
VE14578 (G61)	Cm	Human stool; Oklaoma, USA; 1994	M; T	275	T2	-/+
VE14794 (CE22)	F	Coat Cheese; Corse, France; 1982	-	279	T5	-/+
VE14820	C	Endocarditis; France	E; G; K; S	281	T5	-/+
VE18245 (HH22)	C	Urine; Houston, USA; 1981	A; G; E; T	6	T2	-/+[g]
MMH594	C	Blood; Wisconsin, USA; 1985	E; G; K; S	6	T2	-/+
VE14937 (Symbioflor 1)	P*	Healthy human, Germany, 1950s	-	248	T1	-/+[g]
VE14505	-	Unknown[h]; <1950	-	25	T1	-/+
VE14513	C	Urine; Loir-et-Cher, France	-	168	T2	-/+
VE14518	C	Deep pus; Ardèche, France	-	145	T1	+/+
VE14565	C	Endocarditis; USA; <1992	M; T	55	T1	+/+
VE14573	Cm	Human stool; Oklahoma, USA; 1994	M; T	274	T1	+/+
VE14583	C	Lung; Bouches du Rhône, France	C; E; M; T	276	T1	+/+
VE14596	C	Valve's body; Rhône, France; 1999	M; T	40	T1	+/+
VE14605	S	Human stool; Yvelines, France; 2004	-	86	T5	-/+
VE14668	C	Urine; Yvelines, France	C; E; G; K; M; S; T	6	T2	-/+
VE14675	C	Deep Pus; Yvelines, France	C; E; G; K; M; T	159	T1	-/+
VE14724	Cm	Human stool; Doubs, France; 2003	-	278	T1	+/+
VE14726	Cm	Human stool; Doubs, France; 2003	-	206	T2	-/+
VE14807	Cm	Human stool; Yvelines, France; 2005	-	35	T2	+/+
VE14816	C	Endocarditis; France	E; G; K; M; T	16	T2	+/+
VE14817	C	Endocarditis; France	C; K; M; T	280	T2	+/+
VE14818	C	Endocarditis; France	-	97	T1	-/+

VE14821	C	Endocarditis; France	-	209	T5	-/+
VE14822	C	Endocarditis; France	-	282	T5	+/+
VE14824	F	Cheese; France; 2005	E; M; T	21	T1	+/+
VE14828[f]	F	Cheese; France; 2005	C; E; K; M; T	283	T1	-/+
VE14842 [f]	F	Cheese; France; 2005	E; K; M; T	23	T2	-/+
VE14843 [f]	F	Cheese; France; 2005	K; M; T	133	T1	-/+
VE14844 [f]	F	Cheese; France; 2005	C; E; K; M; T	284	T1	+/+
VE14845 [f]	F	Cheese; France; 2005	C; E; M; T	285	T1	+/+
VE14870 [f]	F	Cheese; France; 2005	K; M; T	9	T5	-/+
VE14878 [f]	F	Cheese; France; 2005	E; K; M; T	26	T1	-/+
VE14893	C	Endocarditis; Doubs; France; 2004	-	286	T2	+/+
VE14927	C	Endocarditis; Ile et vilaine, France	E	288	T1	-/+
VE14929	C	Endocarditis; Ile et vilaine, France	C; E; K; M; S; T	41	T1	-/+
Ef230[i]	S	Urban sewage, Poland	V	nd	nd	nd
Ef423[i]	S	Urban sewage, Poland	V	nd	nd	nd
Ef546[i]	S	Urban sewage, Poland	-	nd	nd	nd
		E. faecium				
VE14727 (BM4147)[j]	C	Patient stool; France; 1986	V;T	95/CC22	nd	nd
VE14141 (TX0016)[j]	C	Blood from endocarditis patient ; Houston; 1992	K; E; Sm, T	18	nd	nd
VE14976 (06-007)[j]	C	French national reference center; France; 2006	Va	21	nd	nd
VE14977 (06-046)[j]	C	French national reference center; France; 2006	Va	64	nd	nd
VE14978 (06-047)[j]	C	French national reference center; France; 2006	Va	78	nd	nd
VE14980 (06-084)[j]	C	French national reference center; France; 2006	Va	18	nd	nd
VE14981 (07-018)[j]	C	French national reference center; France; 2007	Vb	203	nd	nd
VE14982 (07-079)[j]	C	French national reference center; France; 2007	Vb	202	nd	nd
VE14983 (07-095)[j]	C	French national reference center; France; 2007	Va	17	nd	nd
VE14984 (08-225)[j]	C	French national reference center; France; 2008	Vb	192	nd	nd
VE14988 (09-122)[j]	C	French national reference center; France; 2009	Va	280	nd	nd
VE14989 (Aus0004)[j]	C	French national reference center; Australia; 1998	Vb	17	nd	nd

a: C, Cm, F, and S correspond to clinical, commensal, food and sewage origin, respectively. P* indicates one isolated colony from a probiotic concoction of ten *E. faecalis* isolates. – indicates unknown origin. b: A, C, E, F, G, K, M, R, S, Sm, T and V indicate resistance to ampiciliin, cotrimoxazole, erythromycin, fusidic acid, gentamicin, kanamycin, minocycline, rifampicin, sulfamethoxazole/trimethoprim, streptomycin, tetracycline and vancomycin, respectively. Va and Vb indicate the gene involved in vancomycin-resistance. c: Sequence Type. nd indicates not documented. d: Capsule Type. nd indicates not documented. e: CRISPRs indicates the CRISPR1/CRISPR2-group assigned from PCR CRISPR-specific amplification. – indicates no detection. nd indicates not documented. f: Isolates for which some details have been ever given in [52]. g: CRISPRs data were obtained using [24,51]. h: The origin of the type strain is contradictory in the literature. i: :Isolates graciously provided by Dr. Sylwia Bloch and Dr. Alicja Węgrzyn from Department of Molecular Biology, and University of Gdańsk, Wita Stwosza 59, 80-308 Gdańsk, Poland. These strains allows the propagation of podophage vB_Efae230P-4 [44] homologous to Idefix. j : Isolates initially graciously provided by Pr. Vincent Cattoir from CNR souches Enterocoques, Rennes, France.

Table S3: Respective genome accession numbers of the *Enterococcus Picovirinae* phages

Enterococcus Picovirinae phages	Genome accession number
Enterococcus faecalis phage Idefix	LT630001.1
Enterococcus faecalis phage vB_EfaP_IME195	NC_028693
Enterococcus faecalis phage vB_Efae230P-4	NC_025467
Enterococcus faecium phage vB_EfaP_IME199	KT945995.1

Table S4: Features of Idefix ORFs and predicted functions of their products

ORF	Start-End position<br>Translation initiation region*	Amino acids residues	Pi	Best BLASTp similarity with *Enterococcus* phages vB_EfaP_IME195 [a] or vB_Efae230P-4 [b] (E value ; % identity)	Predicted function (Eventual Comments)
**IDF_00**	197-355<br>AAATTTAAGGAGGGAACAAC**ATG**	52	4.94	[a]YP_009191319.1<br>(9e-28 ; 98%)	Hypothetical protein
**IDF_01**	370-900<br>ATCTAAGGAGGAAAATAAAA**ATG**	176	4.65	[a] YP_009191320.1<br>(4e-68 ; 67%)	Hypothetical protein
**IDF_02**	1041-1418<br>ACAGTAGAGGAGAATTTATT**ATG**	125	9.43	[b] YP_009103979.1<br>(5e-57 ; 89%)	Single-stranded DNA binding protein
**IDF_03**	1486-1650<br>TATATCAGGAGGTAACAACA**ATG**	54	4.06		Hypothetical protein
**IDF_04**	1663-1863<br>TGTTTTAAGGAGGAAACAAA**ATG**	66	4.53		Hypothetical protein
**IDF_05**	1847-2269<br>CTACAGAGAGGAGTATATTA**ATG**	140	10.2	[a] YP_009191326.1<br>(2e-96 ; 97%)	Hypothetical protein<br>(putative terminal protein)
**IDF_06**	2262-3503<br>AGCTTATCAGGAGGGTTACA**ATG**	413	6.22	[a] YP_009191327.1<br>(0.0 ; 99%)	Encapsidation protein
**IDF_07**	3516-5864<br>TCAGTTAGGAGTTTTTACAC**ATG**	782	5.68	[b] YP_009191328.1<br>(0.0 ; 98%)	DNA polymerase<br>(B type, protein priming subfamily)
**IDF_08**	5924-6094<br>GGTTAAGGGAGGAATTAATC**ATG**	56	5.04	[a] YP_009191329.1<br>(1e-32 ; 100%)	Hypothetical protein
**IDF_09**	6094-6303<br>GTAACGGAGGTAGAAGCATA**ATG**	69	7.98	[a] YP_009191330.1<br>(7e-23 ; 65%)	Hypothetical protein
**IDF_10**	6294-6467<br>TGTACGGAGGTACCAATTCA**ATG**	57	4.32	[b] YP_009103971.1<br>(8e-30 ; 91%)	Hypothetical protein
**IDF_11**	6468-6701<br>AGAAATTGGAGGATCTTTAA**ATG**	77	4.73	[a] YP_009191332.1<br>(1e-26 ; 71%)	Hypothetical protein
**IDF_12**	6698-7201<br>GAACTATCGGAGGAATTAAA**ATG**	167	9.92	[a] YP_009191333.1<br>(2e-117 ; 97 %)	HNH homing endonuclease
**IDF_13**	7194-8612<br>AATAAGAAAGGGTGTTGCTA**ATG**	472	4.28	[a] YP_009191334.1<br>(0.0 ; 97%)	Endolysin<br>(PlyCA like, putative peptide LysIA<br>of a multimeric endolysin)
**IDF_14**	8695-9051<br>TGGTATTATTAAGTTCTTAA**ATG**	118	9.25	[b] YP_009103967.1<br>(2e-58 ; 83%)	Hypothetical protein
**IDF_15**	9843-9067<br>ATCCGAAGGGAGGTGAATAA**ATG**	258	7.67	[a] YP_009191336.1<br>(0 ; 96%)	Endolysin<br>(N-acetylmuramoyl-L-alanine amidase AmdI)
**IDF_16**	10236-9844<br>AACAAAAGGAGAAACGTTTA**ATG**	130	5.81	[a] YP_009191337.1<br>(5e-89 ; 99%)	Holin
**IDF_17**	11990-10236<br>GATAGAGGGGATTTTCTAAT**ATG**	584	6.79	[a] YP_009191338.1<br>(0.0 ; 93%)	Tail protein
**IDF_18**	12777-11992<br>ACTTACTAGGAGGAGATACA**ATG**	261	4.90	[a] YP_009191339.1<br>(0.0 ; 95%)	Hypothetical protein
**IDF_19**	14354-12789<br>CATGGTAAAGGAGAGAAAAT**ATG**	521	5.27	[a] YP_009191340.1<br>(0.0 ; 98%)	Putative virion protein<br>(calcineurin-like phosphoesterase motif)
**IDF_20**	15032-14367<br>GTTAGGTGGTGAACAACAAA**ATG**	221	4.56	[a] YP_009191341.1<br>(7e-126 ; 98%)	Lower collar protein
**IDF_21**	15999-14965<br>TATAACAAGGAGGTATTTAA**ATG**	344	4.70	[a] YP_009191342.1<br>(0.0 ; 98 %)	Head-tail connector
**IDF_22**	17229-16015<br>CCTAGGAGGAGAAGAATAAT**ATG**	404	5.02	[a] YP_009191343.1<br>(0.0 ; 96%)	Major head protein
**IDF_22.1**	17401-17231<br>AATAAGGAGAGTGCTAAGAA**ATG**	56	4.54	[b]YP_009103958.1<br>(2e-31 ; 96%)	Hypothetical protein
**IDF_23**	17855-17496<br>CTTGACTATTTTACTATATT**ATG**	119	3.27	[b] YP_009103957.1<br>(3e-49 ; 90%)	Hypothetical protein

*The putative RBS sequences are underlined and the start codons are indicated in bold

Table S5. Spontaneous mutations in *epaX* (*ef2170*) or 2169 in pp6$^-$ and resistance to Idefix infection

Clone	ORF<br>Position	Mutation type	Reference	Mutation	Amino acids change	Resistance to Idefix<br>% adsorption (sd)
VE18306 B7	*epaX*<br>2079401	IS		IS256<br>Opposite direction	Frameshift	+<br>9.14 (7.3)
VE18306 B10	*epaX*<br>2079381	IS		IS256<br>Opposite direction	Frameshift	+<br>7.04 (3.0)
VE18306 C6	*epaX*<br>2079493	IS		IS256<br>Same direction	Frameshift	+<br>11.1 (8.3)
VE18306 C7	*epaX*<br>2079432	IS		IS256<br>Same direction	Frameshift	+<br>14.2 (4.9)
VE18306 E1	*epaX*<br>2079518	IS		IS256<br>Same direction	Frameshift	+<br>7.27 (4.0)
VE18306 G12	*epaX*<br>2079402	IS		IS256<br>Opposite direction	Frameshift	+<br>8.29 (6.2)
VE18306 F2	*ef*2169<br>2077970-2078002	D	TGACTTCAGTTATCAGT<br>AATCCAGAAATTAAAA		11 amino acids deleted	+<br>36.17 (6.6)
VE18306 F12	*ef*2169<br>2077970-2078002	D	TGACTTCAGTTATCAGT<br>AATCCAGAAATTAAAA		11 amino acids deleted	+<br>45.4 (5.6)
	2078627-2078637	SNPs	CATAACTAAAT	ACATAACTAAA	5 amino acids substituted	
VE18306 F3	*ef*2169<br>2077970-2078002	D	TGACTTCAGTTATCAGT<br>AATCCAGAAATTAAAA		11 amino acids deleted	+<br>28.3 (3.9)
	2078898	SNP	A	C	Tyr/Stop	

IS: Insertion Sequence, D: Deletion, SNP: Single Nucleotide Polymorphism
